# The rumen microbiota and metabolism of dairy cows are affected by the dietary rate of inclusion of *Yucca schidigera* extract

**DOI:** 10.1128/spectrum.00641-25

**Published:** 2025-06-12

**Authors:** Timothy J. Snelling, Marykate Condren, James A. Huntington, Helen E. Warren, Jules Taylor-Pickard, Liam A. Sinclair

**Affiliations:** 1 Animal Science Research Centre, Department of Agriculture and Environment, Harper Adams University 6000, Newport, United Kingdom; 2 Alltech Bioscience Centre, Dunboyne, Ireland; Cleveland Clinic Lerner Research Institute, Cleveland, Ohio, USA

**Keywords:** ammonia, microbiome, nitrogen

## Abstract

Natural plant compounds can be used to supplement livestock diets, improving feed efficiency, production, and health, while also reducing environmental impact. In the present study, a *Yucca schidigera* (Mohave Yucca) extract was added at four rates of inclusion (ROI) of 0, 5, 15, or 30 g/day to a ryegrass and maize silage-based diet and fed to dairy cows in a 4 × 4 Latin square experimental design. Each period was 28 days in duration, with sampling undertaken during the final week of each period. Solid phase digesta (SPD) and liquid phase digesta (LPD) samples were collected via a rumen cannula and analyzed for volatile fatty acids (VFAs), ammonia N, and microbiome using 16S rRNA gene sequencing. Total fecal and urine collection was undertaken over a 3-day period. Rumen microbial diversity was not affected by ROI (LPD: *P* = 0.180; SPD: *P* = 0.059). However, discriminant analysis found a decrease in *Methanobrevibacter millerae* (linear discriminant analysis, LDA = 2.15) and an increase in an unclassified species of Proteobacteria (LDA = 2.10) associated with ROI. Univariate analysis also revealed differential abundance of operational taxonomic units classified as *Prevotellaceae* and *Fibrobacteraceae* by ROI (*P* < 0.05). Maximum rumen ammonia N concentration decreased linearly from 228 to 109 mg/L with ROI (*P* = 0.044). Rumen VFA concentration was unaffected with the exception of propionate, which showed a linear increase with ROI (*P* = 0.010). The diurnal rumen pH range (maximum–minimum) also decreased with ROI (*P* = 0.004). Dry matter intake and milk yield were not affected (*P* > 0.05) by ROI; however, there was a linear increase in milk fat content from 38.9 to 42.0 g/kg with ROI (*P* < 0.05).

**IMPORTANCE:**

Domestic livestock such as dairy cows are inefficient utilizers of dietary nitrogen. This increases feed costs and reduces animal production efficiency. Excreted nitrogenous compounds are also an environmental hazard, such as when they enter water courses as nitrate or are lost to the atmosphere as ammonia or nitrous oxide. Dietary protein is degraded in the rumen via the activity of the microbial population, mainly into ammonia, which may then be utilized by the microbial population to synthesize microbial protein or absorbed into the blood and potentially excreted. Manipulation of the diet or altering the microbial population may increase the utilization of dietary protein, increasing animal performance, decreasing feed costs, and reducing the environmental impact of milk production. This study examines the effect of *Yucca schidigera* extract on the rumen microbiome and nitrogen utilization in dairy cows.

## INTRODUCTION

The diversity and metabolic activity of the rumen microbial community are significantly affected by intake and dietary composition (1–3). In high-production ruminant livestock systems, diets are often fed as a total mixed ration (TMR) with the ratio of forage and high-energy concentrate feeds and composition of micronutrients balanced to meet the nutrient requirements for animal production (4). The inclusion of supplements derived from naturally occurring plant extracts, including polyphenolic compounds or saponins, has also become widespread to modulate the composition and functional activity of the rumen microbiome to improve feed efficiency, health, and reduce the environmental impact (1).

*Yucca schidigera* (*Y. schidigera*, Mohave Yucca) is a desert plant native to the southwestern United States and Mexico (5). The capacity to bind ammonia has led to its use in the pig and poultry industries to reduce the toxic odor from manure and improve animal performance (6, 7). *Y. schidigera* has also been found to have an effect on microbial growth, with saponin compounds extracted from the stem of the plant having potent anti-yeast activity (5). *In vitro*, *Y. schidigera* extract binds ammonia and affects the growth of microbes, including inhibition of the fiber-degrading *Butyrivibrio fibrisolvens* and stimulation of a *Prevotella ruminicola* strain (8). In a pure culture system, the addition of *Y. schidigera* extract to the growth medium had no apparent effect on the growth of cellulolytic ruminal bacteria *Ruminococcus flavefaciens*, *Fibrobacter succinogenes,* and *Ruminococcus albus* but reduced endoglucanase activity (9). In the same study, *Ruminobacter amylophilus*, *Prevotella bryantii,* and *Streptococcus bovis* growth curves were reduced, whereas *Selenomonas ruminantium* growth was enhanced when *Y. schidigera* was included in the growth medium. Ciliate protozoa activity has also been demonstrated to be reduced by the addition of *Y. schidigera* extract compared to other saponin sources (8).

*In vivo*, dietary inclusion of *Y. schidigera* did not have any effect on rumen degradability in heifers, although the reported increase in propionate concentration could have been a consequence of a selective effect of the *Y. schidigera* on microbial activity, while the reduction in rumen ammonia N concentration was most probably a result of direct binding as well as decreased protein degradation by rumen ciliates (10). A tendency for reduced rumen ammonia N concentration was also found in steers fed diets with and without urea supplementation when *Y. schidigera* was fed, with a concurrent reduction of total volatile fatty acid (VFA) concentration and the acetate:propionate ratio (11). At lower doses, *Y. schidigera* extract has not been found to have any detrimental effect on cow performance, health, or rumen digestibility of organic matter and dietary fiber (12), and at a rate of inclusion (ROI) of 5 g/day, some alteration of the rumen microbiome, including decrease of a species of *Methanobrevibacter* and change in abundance of various species of *Prevotella,* was observed (13). Similarly, supplementing *Y. schidigera* to the diet did not affect milk yield (13). However, other studies have reported a trend for a decrease in dry matter intake (DMI) associated with a higher ROI (14). Apart from these few studies, little work has been undertaken directly comparing different ROI of *Y. schidigera* on the rumen microbiome, metabolism, urinary and fecal nitrogen excretion, and performance in dairy cows. The aim of the current study was to determine the effects of *Yucca schidigera* ROI on the rumen microbiome, metabolism, and nitrogen use efficiency in lactating dairy cows when added to a maize and grass silage-based total mixed ration.

## MATERIALS AND METHODS

### Animals and experimental design

The study was conducted over a 16 week period from January to April 2022 at the Harper Adams University Dairy Cow Metabolism Unit (Shropshire, UK). The experimental design was a Latin square, with four periods each of 4 weeks duration, with measurements undertaken during the final week of each period.

Four multiparous Holstein-Friesian dairy cows with a liveweight (mean ± SE) of 712 ± 48.6, body condition score (15) of 2.9 ± 0.24, and milk yield of 40 ± 2.0 kg/day at the start of the study and had previously been fitted with 10 cm diameter permanent rumen cannula (Bar Diamond, Idaho, USA) were used. The cows were loose housed in a sawdust-bedded pen that provided 17.5 m^2^/cow living space and contained a concrete feed area that was manually scraped twice a day. During the sample collection periods, the cows were housed in individual metabolism stalls fitted with foam mattresses for 6 days to allow rumen, fecal, and urine sample collection. Cows had continuous access to water at all times.

### Forages and diets

All cows received a TMR containing first-cut grass silage and maize silage (40:60 DM basis) and straight feeds (Table S1) via individual feed bins (American Calan, Northwood, NH, USA). The TMR was formulated according to Thomas (16) for a milk production of 38 kg/cow/day and was fed at 105% of the previous recorded intake. Feed out took place daily at 0800 h, with refusals collected each morning during the sampling week.

The basal diet was unsupplemented or supplemented with dried and chopped, whole *Yucca schidigera* extract (De-Odorase, Alltech, Nicholasville, KY, USA) to produce four dietary treatments:

- 0: control diet, no supplement.
- 5: control diet, plus 5 g/cow/day *Yucca schidigera.*
- 15: control diet, plus 15 g/cow/day *Yucca schidigera.*
- 30: control diet, plus 30 g/cow/day *Yucca schidigera.*

### Sampling

Rumen digesta samples were collected on day 4 of the final week of each period at 0800 h (immediately before morning feeding) and then at 3 h intervals during the day (1100, 1400, 1700, and 2000 h) (13). Two grab samples of digesta were collected via the cannula at approximately 20 and 80 cm depth. The two samples were pooled and strained through four layers of cheese cloth to separate the solid phase digesta (SPD) from the liquid phase digesta (LPD). Rumen pH was recorded immediately after sampling using a subsample of the LPD. A subsample of the LPD (45 mL) was then added to a solution of HPO_3_ (5 mL of 25%, wt/vol) and stored at −20°C for subsequent analysis of VFA and ammonia N. For microbial community analysis, LPD samples collected at 1100, 1400, and 1700 h (10 mL) were added to tubes prefilled with 10 mL of 30% (vol/vol) glycerol solution. The corresponding SPD samples (~50 g) were placed in a 100 mL sample pot, and 50 mL of 15% (vol/vol) glycerol solution was added. Both the LPD and SPD samples were then stored at −20°C.

For three 24 h periods during the sampling week, total urine output was collected using a modified 2 L catheter bag (Optimum Medical Solutions, West Yorkshire, UK) fitted with a pipe (32 mm internal diameter) leading to a barrel (25 L) (13). The catheter bags were fitted around the vulva of the cow with Velcro applied with a contact adhesive. The urine was acidified by the addition of 0.5 L of 50% sulfuric acid. After a 24 h collection period, the total volume was weighed, and subsamples were stored at −20°C for subsequent analysis. Fecal samples were also collected during the same time period by collecting the freshly deposited material at approximately 2–3 h intervals post-feedout (17), pooling, mixing, subsampling, and storing at −20°C prior to analysis.

### Microbial community analysis

Solid phase digesta samples (~50 g) were washed twice in 1 L of 0.9% (wt/vol) saline solution to remove residual LPD-associated microbes (18). The SPD sample (100 g) was then transferred to a strainer bag with an additional 100 mL saline solution and homogenized in a stomacher (Seward, West Sussex, UK) at 230 rpm for 5 min to detach fiber-associated microbes. Both the supernatant from this process (SPD) (15 mL) and LPD samples (5 mL) were centrifuged (8,500 × *g* for 20 min) separately to obtain two microbial pellets per digesta sample (0.5 g). These were transferred to 2 mL screw top tubes for DNA extraction by repeated bead beating and column purification (Qiagen UK) based on Yu and Morrison (19).

Amplification of the V4 region of the rRNA gene was carried out by PCR in triplicate (25 µL) using High-Fidelity DNA polymerase (Q5, NEB Inc.) and barcoded universal prokaryotic primers (20). The PCR products were cleaned and quantified using the Quant-iT PicoGreen dsDNA Assay Kit (Invitrogen). The samples were pooled in equimolar quantities and run on a 1% (wt/vol) agarose/TBE gel to separate residual primers and dNTPs. The band containing the amplicons was excised and purified (Wizard SV Gel and PCR Clean-Up System-Promega). The libraries were quality assessed using an Agilent 2100 Bioanalyzer System and sequenced using the Illumina MiSeq v2 250 paired-end reagent kit at Edinburgh Genomics (University of Edinburgh, UK). Sequence data were analyzed using mothur 1.44.0 (21) with steps to assemble paired-end reads and remove low-quality and chimeric sequences and were clustered into operational taxonomic units (OTUs) at 97% identity (File S1). Taxonomic classification of the representative sequences was conducted using the SILVA 132 SEED reference database (22). Selected OTUs were also classified using BLASTn against type material from the current NCBI reference database. Prior to the diversity and statistical analysis, libraries were normalized by subsampling to 10,200 reads per sample, and low-abundance OTUs (total number of reads per OTU <10) were removed from the data set. This resulted in a total of 1,631 OTUs at 97% sequence identity across all the samples, which were numbered in order of abundance by sequence counts (File S2).

### Chemical analysis

Forage and TMR samples were bulked between days for each period, and the subsamples were analyzed using Association of Official Analytical Chemists (23) methods for dry matter (943.01), crude protein (CP) (990.03), and ash (AOAC 942.05). Neutral detergent fiber (NDF) was determined based on Van Soest et al. (24) and expressed exclusive of residual ash. Milk composition was analyzed by National Milk Laboratories (NML, Wolverhampton, UK) for fat, protein, lactose, and urea using near and mid-infrared spectroscopy (Foss, Denmark).

Volatile fatty acids were analyzed in the liquid rumen fluid fraction by GC using methods based on Erwin et al. (25) using a DB-FFAP column (30 m × 250 µm × 0.25 µm) (Agilent 6890, Stockport, UK). Column temperature was 60°C, and nitrogen was used as the carrier gas with a flow rate of 2 mL/min. The S/SL inlet temperature was 250°C with a split ratio of 20:1 and a split flow rate of 40 mL/min. The flame ionization detector temperature was 300°C, hydrogen flow to the flame jet was 40 mL/min, and air flow to the detector chamber was 450 mL/min. Symmetrical peaks of the VFA and isomers were eluted in a run time of 10 min, and concentrations were calculated against a standard curve for each compound based on the peak area. For rumen LPD, urine, and fecal samples, the total and ammonia N concentration was determined from a method adapted from MAFF (26) using an auto-titrator (FOSS, Warrington, UK, and Buchi Labortechnik AG, CH-9230, Flawil, Switzerland).

### Intake and milk parameters

Intake was recorded daily during the sampling week of each period. Samples of the grass and maize silage were collected weekly, dried at 105°C, and the dietary amounts were altered to achieve the desired ratio (DM basis). The TMR samples were collected daily during the sampling week of each period and stored at −20°C for subsequent analysis. The cows were milked twice daily at 0600 and 1600 h, and the yield was recorded. During the sampling week of each period, six milk samples were collected (three AM and three PM) for the analysis of fat, protein, lactose, casein, and urea (NML laboratories, Wolverhampton, UK). Body weight and condition scores were recorded prior to beginning and at the end of each sampling period.

### Statistical analysis

Data were analyzed using GenStat Release 20.1 (VSN International Ltd). Performance data were analyzed as a Latin square design using the model: *Y* = *μ* + *T*_*i*_ + *P*_*j*_ + *A*_*k*_ + *ε*_*ijk*_, where *Y* is the observation, *μ* is the overall mean, *T*_*i*_ is the treatment, *P*_*j*_ is the fixed effect of period, *A*_*k*_ is the random animal effect, and *ε*_*ijk*_ is the residual error. Treatment degrees of freedom were further split into linear, quadratic, or cubic effects. Data for hourly rumen parameters were analyzed as repeated measurements. Sequence data were analyzed for the depth of coverage per library (27). Microbiome beta diversity was calculated using Bray-Curtis dissimilarity, with the resulting distance matrix used to generate a non-metric multi-dimensional scaling (NMDS) plot with significant differences between ROI determined using analysis of molecular variance with 10,000 iterations. Taxonomic biomarkers associated with *Y. schidigera* extract ROI were determined using linear discriminant analysis (LDA) effect size (LEfSe) (28) with values of *P* < 0.05 and cutoff effect size set at LDA > 2.0. Univariate analysis of filtered sequence counts was also carried out to identify variability of OTU abundance associated with ROI at values of *P* < 0.05.

## RESULTS

### Rumen microbiome

Sequence coverage per sample measured using Good’s statistic was between 98.5% and 99.6% per library. Total relative abundance of taxonomic groups at phylum level was Bacteroidetes (40%), Firmicutes (25%), Spirochaetes (10%), Euryarchaeota (8%), Fibrobacteres (5%), and Proteobacteria (2%), with unclassified bacteria and low abundance phyla (<1%) making up the remaining 10%. As a representation of microbial functional groups, the Firmicutes to Bacteroidetes ratio (F:B) was reduced with 15 g of ROI, although with an interaction with sampling time (*P* = 0.015), with F:B increasing with 0 and 5 g of ROI and decreasing F:B with 15 and 30 g of ROI in the 1400 h timepoint samples (Table 1). No differences or interactions were found in the Archaea to Bacteria ratio (A:B) with ROI. There was no effect (*P* > 0.05) of ROI on alpha diversity within the LPD and SPD samples.

**TABLE 1:** Mean rumen microbial diversity and taxon ratios by *Yucca schidigera* extract rate of inclusion, including interactions with time of sampling^*a*^

	*Yucca schidigera* (g/cow/day)				SED	*P-*values		
	0	5	15	30		ROI	T	R × T
LPD								
OBS	762.8	736.2	752.3	751.4	12.2	0.493	0.081	0.998
Chao1	1,034	1,015	1,025	1,019	20.3	0.913	0.418	1.000
Shannon	4.624	4.561	4.604	4.586	0.048	0.812	0.692	0.842
Inv. Simpson	26.10	25.36	25.57	25.70	1.59	0.989	0.579	0.866
SPD								
OBS	732.6	730.3	767.3	745.1	17.9	0.446	0.096	0.694
Chao1	1,013	993.1	1,064	1,044	21.0	0.120	0.589	0.299
Shannon	4.727	4.765	4.885	4.789	0.64	0.361	0.034	0.806
Inv. Simpson	33.79	34.54	40.99	35.33	2.81	0.273	0.123	0.949
Combined								
A:B ratio	0.082	0.099	0.086	0.078	0.008	0.256	0.432	0.929
F:B ratio	0.636	0.664	0.582	0.643	0.017	0.008	0.720	0.015

^*a*^ Combined, data pooled from LPD and SPD; T, time of sampling; R × T, interaction between rate of inclusion and time of sampling; and SED, standard error of difference.

**Fig 1:**
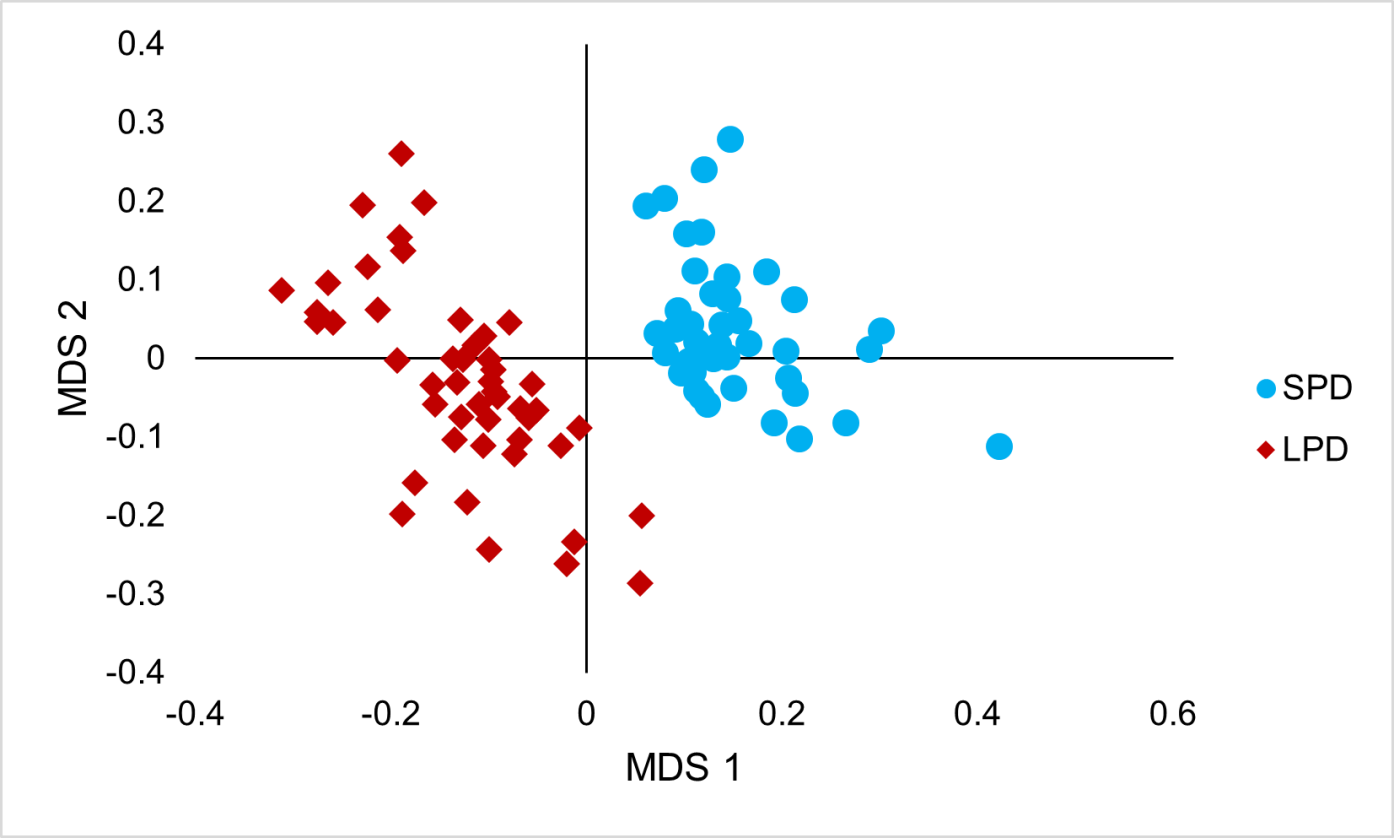
NMDS plot of microbial communities from SPD and LPD samples. Stress = 0.17. PERMANOVA 10,000 iterations: *F*-value: 54.6, *R*^2^: 0.39, and *P* = 0.001.

Bray-Curtis dissimilarity at the OTU level revealed very strong clustering of the microbiomes within each of the LPD and SPD samples (*P* < 0.001; Fig. 1) and were therefore treated as two separate data sets for downstream analysis. No clustering by *Y. schidigera* extract ROI was found in either LPD (*P* = 0.180) or SPD samples (*P* = 0.059). However, biomarkers were detected at LDA > 2.0 with a *Methanobacteriaceae* species (OTU0004) and an unclassified bacterial species (OTU0005) in the LPD samples and *Spirochaetaceae* species (OTU0002) in the SPD samples (Table 2). OTU0004 decreased with ROI (*P* = 0.047), and OTU0005 increased with ROI (*P* = 0.092). Univariate analysis based on mean normalized sequence counts found OTUs classified to *Prevotellaceae*, *Fibrobacteraceae,* and *Spirochaetaceae* families that differed in relative abundance by *Y. schidigera* extract ROI in both LPD and SPD samples (Table 3).

**TABLE 2:** Biomarkers (OTUs) determined using LEfSe scores > 2.0 with mean filtered sequence counts associated with *Yucca schidigera* rate of inclusion

	*Yucca schidigera* (g/cow/day)					Taxonomic classification (% identity)	
	0	5	15	30	LDA	SILVA 132 SEED	BLASTn Type Strain
LPD							
OTU0004	767	896	752	617	2.15	*Methanobacteriaceae*	*Methanobrevibacter millerae* strain ZA-10 (99)
OTU0005	593	575	665	822	2.10	Bacteria (unclassified)	*Gilliamella intestini* strain LMG 28358 (85)
SPD							
OTU0002	912	1,034	834	995	2.00	*Spirochaetaceae*	*Treponema bryantii* strain RUS-1 (98)

**TABLE 3:** Univariate analysis (ANOVA) of mean filtered sequence counts^*a*^

	*Yucca schidigera* (g/cow/day)					Taxonomic classification (% identity)	
	0	5	15	30	*P-value*	SILVA 132 SEED	BLASTn Type Strain
LPD							
OTU0009	185.9	181.8	234.8	191.7	0.039	*Prevotellaceae* (100)	*Prevotella ruminicola* strain Bryant 23 (96)
OTU0013	113.3	128.2	73.6	107.7	0.024	*Fibrobacteraceae* (100)	*Fibrobacter succinogenes* subsp. *elongatus* strain HM2 (95)
OTU0022	88.0	83.9	98.9	107.3	0.006	*Prevotellaceae* (100)	*Prevotella brevis* strain GA33 (93)
OTU0023	40.0	27.0	26.3	29.6	0.030	*Fibrobacteraceae* (100)	*Fibrobacter succinogenes* subsp. *succinogenes* S85 (97)
SPD							
OTU0013	93.4	126.0	75.0	137.11	0.012	*Fibrobacteraceae* (100)	*Fibrobacter succinogenes* subsp. *elongatus* strain HM2 (95)
OTU0020	68.5	61.2	87.1	52.8	0.047	*Prevotellaceae* (100)	*Prevotella ruminicola* strain Bryant 23 (92)
OTU0021	100.6	126.1	89.2	114.8	0.047	*Spirochaetaceae* (89)	*Treponema bryantii* strain RUS-1 (96)
OTU0022	23.8	32.4	43.1	35.0	0.029	*Prevotellaceae* (100)	*Prevotella brevis* strain GA33 (93)

^*a*^ OTUs (in order of relative abundance) with variable sequence counts by *Yucca schidigera* extract rate of inclusion (*P* < 0.05).

### Rumen metabolism

**Fig 2:**
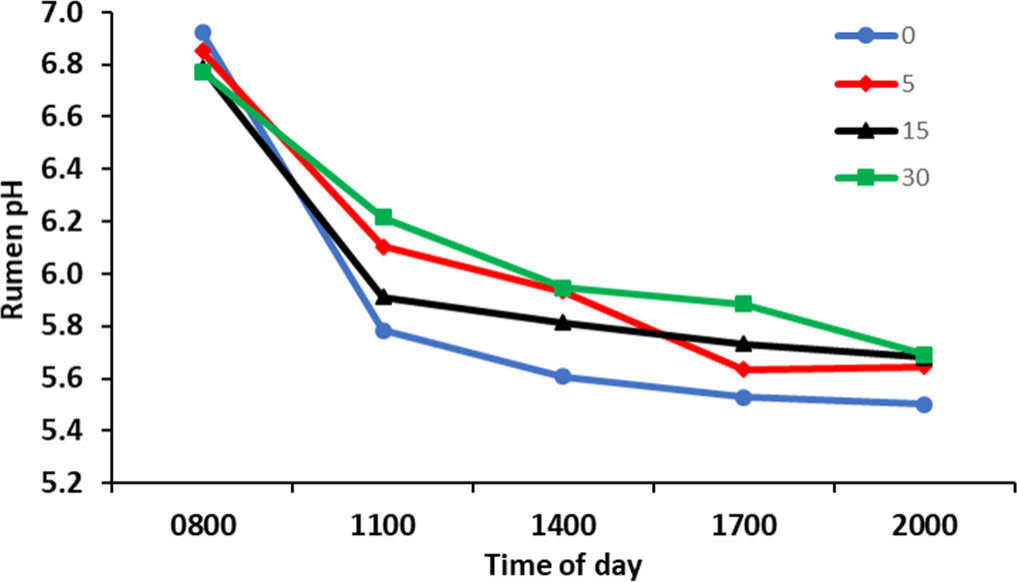
Rumen pH at sampling times during the day by *Yucca schidigera* extract rate of inclusion. Pooled standard error of difference = 0.17. Time, *P* < 0.001; treatment × time, linear *P* = 0.041; quadratic *P* = 0.77; and cubic *P* = 0.305. Cows were fed at 0800 h.

Rumen pH was highest at 0800 h, immediately prior to feeding, and decreased post-feeding to reach a nadir at 2000 h (*P* < 0.001; Fig. 2). There was an interaction (*P* < 0.05) between time and treatment at 1700 h, with rumen pH being highest in cows receiving 30 g/day of *Y. schidigera* extract and lowest in those receiving no supplement. The diurnal range in rumen pH had an inverse linear correlation with *Y. schidigera* extract ROI (*P* < 0.01; Table 4). There was, however, no relationship between ROI (*P* > 0.05) and mean, maximum, or minimum rumen pH.

**TABLE 4:** Effect of the dietary level of *Yucca schidigera* extract rate of inclusion on rumen pH, VFA (mM), ammonia (mg/L), and urine nitrogen (g/day) in dairy cows

	*Yucca schidigera* (g/cow/day)					*P*-value^*a*^		
	0	5	15	30	SED^*b*^	Lin	Quad	Cubic
Mean rumen pH	5.87	6.03	5.99	6.10	0.119	0.154	0.803	0.284
Maximum rumen pH	6.92	6.85	6.79	6.77	0.123	0.275	0.576	0.912
Minimum rumen pH	5.46	5.57	5.58	5.66	0.122	0.179	0.804	0.595
Max-min rumen pH	1.46	1.28	1.21	1.11	0.075	0.004**	0.215	0.316
Rumen total VFA (mM)	95.5	93.1	91.6	95.3	3.04	0.935	0.183	0.936
Proportion of total VFA								
Acetate	0.657	0.663	0.655	0.656	0.0029	0.253	0.923	0.035*
Propionate	0.183	0.183	0.189	0.191	0.0024	0.010**	0.641	0.254
Butyrate	0.128	0.123	0.124	0.121	0.0030	0.100	0.721	0.298
Iso-butyrate	0.006	0.006	0.006	0.006	0.0002	0.827	0.214	0.922
Valerate	0.014	0.014	0.014	0.014	0.0004	0.250	0.812	0.537
Iso-valerate	0.012	0.011	0.012	0.011	0.0015	0.959	0.974	0.533
Ac:prop ratio	3.63	3.66	3.51	3.47	0.055	0.010**	0.687	0.188
(Ac + but):prop ratio	4.33	4.34	4.18	4.11	0.073	0.010**	0.645	0.382
Rumen mean NH_3_ (mg/L)	123	109	108	109	8.9	0.257	0.255	0.381
Rumen max NH_3_ (mg/L)	228	195	183	179	17.2	0.044*	0.162	0.429
Rumen min NH_3_ (mg/L)	55	47	53	43	6.4	0.195	0.628	0.192
Rumen max-min NH_3_ (mg/L)	173	148	130	137	18.9	0.118	0.155	0.795
Ingested N (g/day)	642	641	652	652	22.8	0.684	0.558	0.561
Milk N (g/day)	174	165	167	171	10.1	0.967	0.849	0.908
Fecal total N (g/day)	222	194	197	215	10.8	0.886	0.037*	0.026*
Urine total N (g/day)	172	156	176	160	4.8	0.629	0.695	0.131
Retained N (g/day)	73	126	111	105	19.7	0.921	0.215	0.241

^*a*^ *P < 0.05 and **P < 0.01.

^*b*^ SED, standard error of difference.

**Fig 3:**
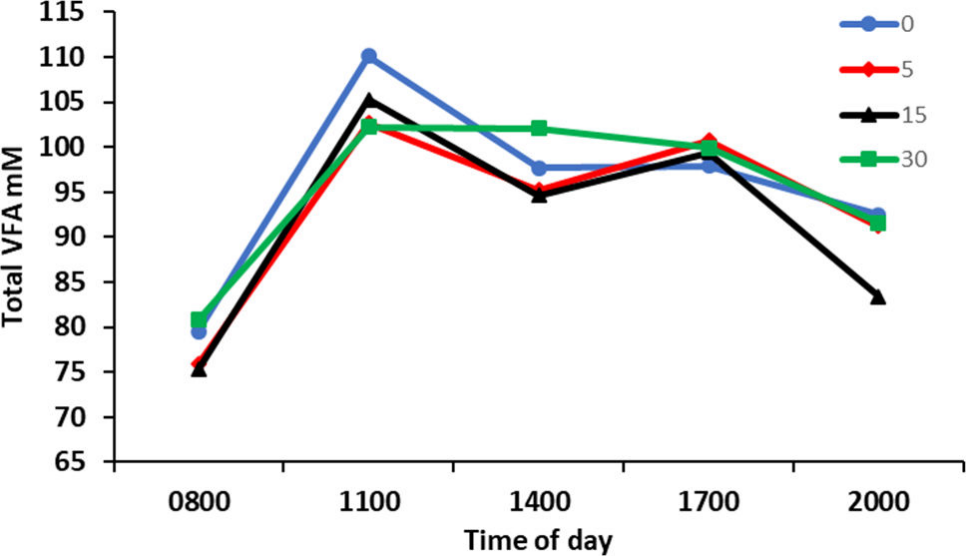
Rumen total VFA concentration at sampling times during the day by *Yucca schidigera* extract rate of inclusion. Pooled standard error of difference = 6.5. Time, *P* < 0.001; treatment × time, linear *P* = 0.698; quadratic *P* = 0.730, and cubic *P* = 0.739. Cows were fed at 0800 h.

**Fig 4:**
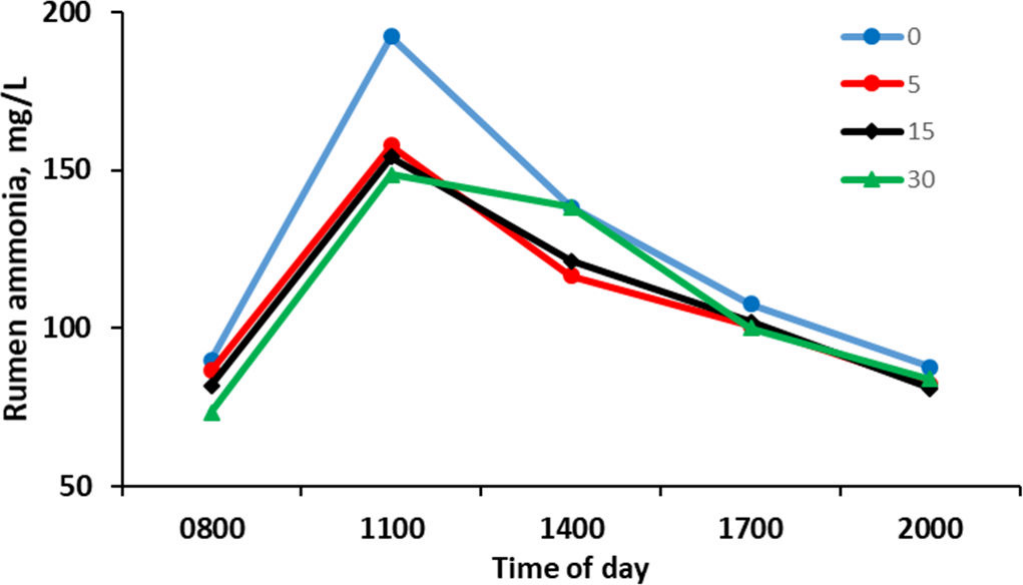
Rumen total ammonia N concentration at sampling times during the day by *Yucca schidigera* extract rate of inclusion. Pooled standard error of difference = 39.7. Time, *P* = 0.017; treatment × time, linear *P* = 0.847; quadratic *P* = 0.847, and cubic *P* = 0.960. Cows were fed at 0800 h.

There was no effect (*P* > 0.05) of ROI of *Y. schidigera* extract on the mean rumen VFA concentration (Table 4), but there was an effect of time (*P* < 0.05), with an increase in VFA concentration from 0800 h to a maximum at 1100 h, followed by a gradual decrease with the remaining samples to 2000 h (Fig. 3). There was no effect (*P* > 0.05) of ROI of *Y. schidigera* extract on the rumen fluid proportion of acetate, isobutyrate, valerate, or iso-valerate. In contrast, the concentration of propionate increased (*P* < 0.05), and butyrate tended (*P* = 0.10) to decrease linearly with *Y. schidigera* extract ROI. The acetate to propionate ratio and the acetate + butyrate to propionate ratio also decreased (*P* = 0.01) with *Y. schidigera* extract ROI. Rumen ammonia N concentration was lowest prior to feeding at 0800 h and reached a maximum 3 h post-feeding, before decreasing (Fig. 4). The maximum rumen fluid ammonia N concentration had a negative linear correlation with *Y. schidigera* extract ROI (Table 4). There was no effect (*P* > 0.05) of the ROI of *Y. schidigera* extract on the mean, minimum, or maximum-minimum concentration of rumen ammonia N.

Quadratic and cubic linear effects were found with the ROI of *Y. schidigera* extract on the fecal total N output, with a lower mean N excretion (g/day) with 5 and 15 g ROI compared to 0 and 30 g ROI. There was no effect (*P* > 0.05) of *Y. schidigera* extract ROI on urine total N output (g/day). There was also no effect of the *Y. schidigera* extract ROI on ingested N, milk N, urine total N, and retained N.

### Dietary chemical composition, intake, and performance

The chemical composition of the basal ration was close to the predicted values (Table S1), with a mean DM, CP, and NDF of 349, 175, and 439 g/kg DM, respectively. The mean DM intake was 23.2 kg/day, and there was no effect (*P* > 0.05) of dietary treatment (Table 5). There was a linear and positive effect of *Y. schidigera* extract ROI on milk fat content (*P* < 0.05), which was 3.1 g/kg higher in cows when fed 30 g/day compared to 0 g/day. There was, however, no effect (*P* > 0.05) of ROI on daily milk fat yield, milk yield, milk protein, and casein or lactose concentration or yield. There was a quadratic effect of ROI of *Y. schidigera* extract on live weight (*P* < 0.05), which was lowest in cows when fed 15 g/day *Y. schidigera* extract, and a trend for a linear effect (*P* = 0.051) on body condition score, being lowest in cows when fed 30 g/day, but there was no effect (*P* > 0.05) on daily liveweight change.

**TABLE 5:** Effect of *Yucca schidigera* extract rate of inclusion on the DM intake and performance of dairy cows

	*Yucca schidigera* (g/cow/day)					*P*-value^*a*^		
	0	5	15	30	SED^*b*^	Lin	Quad	Cubic
Intake (kg DM/day)	22.9	22.9	23.8	23.3	0.65	0.453	0.302	0.421
Milk yield (kg/day)	37.4	35.4	37.6	36.9	1.39	0.815	0.981	0.148
Milk fat (g/kg)	38.9	40.7	40.7	42.0	0.093	0.030*	0.531	0.241
Milk protein (g/kg)	29.8	29.9	29.0	29.7	0.69	0.685	0.378	0.420
Milk casein (g/kg)	24.1	24.3	23.6	24.1	0.49	0.760	0.368	0.365
Milk lactose (g/kg)	44.7	45.2	44.3	45.0	0.31	0.975	0.210	0.049*
Milk urea (mg/dL)	10.2	9.7	13.0	11.4	1.32	0.206	0.174	0.164
Fat yield (kg/day)	1.45	1.42	1.53	1.53	0.083	0.215	0.690	0.434
Protein yield (kg/day)	1.11	1.05	1.09	1.09	0.041	0.989	0.610	0.199
Lactose yield (kg/day)	1.67	1.60	1.66	1.66	0.065	0.811	0.782	0.301
Liveweight (kg)	738	731	721	730	5.0	0.178	0.027*	0.699
Liveweight change (kg/day)	0.55	0.43	0.02	0.29	0.286	0.315	0.175	0.601
Body condition score	3.25	3.13	3.13	3.06	0.068	0.051	0.443	0.257

^*a*^ *P < 0.05.

^*b*^ SED, standard error of difference.

## DISCUSSION

The current study used a 4 × 4 Latin square design with four levels of inclusion of *Y. schidigera* extract and four rumen cannulated dairy cows. This study design has been used widely in recent studies that have investigated dietary effects on rumen fermentation and the microbiome (29–31), although it is recognized that it may be limited by the number of animals per treatment. The *a priori* calculations for the current study indicated that the effect size for changes in rumen fermentation parameters and the microbiome, which was the primary objective, was anticipated to be large due to the high ROI of *Y. schidigera* extract. As a consequence, there was sufficient power to detect several differences in these parameters. Lovett et al. (14) used a 3 × 3 Latin square design with three animals and three rates of inclusion of *Y. schidigera* and also reported significant effects on rumen fermentation parameters and protozoal numbers.

Dietary N for livestock can be provided either in the form of protein or as non-protein N in urea or other nitrogen compounds (4). The majority of dietary protein, peptide, and amino acid undergoes degradation in the rumen by microbial metabolism, ultimately leading to the production of ammonia (32, 33). In dairy production systems, decreasing the excretion of nitrogen compounds can reduce the cost of milk production and help decrease the detrimental effects on the environment and human health (33–35). *Y. schidigera* products are often used to reduce ammonia emissions from manure or slurry (8), and in the current study, a commercial product (De-Odorase) obtained from pulverized stems of *Yucca schidigera Roezl ex Ortgies* (Mojave yucca) that contained a mix of saponins and glycofractions was used as a dietary supplement for high-yielding dairy cows. The supplement was added to a maize and grass silage-based TMR at four rates of inclusion from 0 to 30 g/day to determine the effect on N use, the rumen microbiome, rumen metabolism, and production.

### Rumen microbial metabolism and microbiome

As an indication of rumen microbial metabolism, mean rumen pH followed normal diurnal changes post-morning feedout (13). There was a linear effect of an increased ROI of *Y. schidigera* extract reducing the difference in the maximum and minimum pH in the current study, indicating a more stable rumen pH. This could indicate a potential benefit of *Y. shidigera* as a means to ameliorate subacute ruminal acidosis, which is defined on the basis of both reduced pH and pH variability (36). A reduction in the ratio of acetate to propionate in the rumen fluid in the current study was driven mainly by an increase in the mean proportion of propionate. An increase in rumen propionate concentration with *Y. schidigera* extract supplementation has been reported previously with beef steers (11) and heifers (10).

Comparable to the effect observed in cattle slurry (8), there was a linear reduction of the maximum rumen NH_3_-N concentration with increased ROI at 3 h post-feed out. This time point is often associated with an increase in rumen microbial activity (37) and in the current study was also associated with a decrease in rumen pH. The mechanism of ammonia reduction by *Y. schidigera* extract has been proposed to be due to direct binding of ammonia by the glycol component of saponins (38) or by modifying the rumen microbial composition and/or activity to promote ammonia uptake for rumen microbial protein synthesis (39). Alternatively, rumen ciliate protozoa counts can be reduced by *Y. schidigera*, which affects the breakdown of microbial protein by these microorganisms (8). As rumen NH_3_ accumulates it is absorbed across the epithelium and is transported to the liver, where it is converted into urea. This can then either be recycled back to the rumen directly across the rumen or via saliva or excreted in the urine (40, 41). Despite the decrease in rumen NH_3_ observed here, there was no effect of *Y. schidigera* inclusion on the N balance, including excreted N in the urine or feces.

PERMANOVA of the Bray-Curtis dissimilarity in both LPD and SPD samples did not reveal any significant microbiome clustering associated with *Y. schidigera* extract ROI. However, discriminant analysis identified OTU0004 (*Methanobacteriaceae*) and OTU0005 (Bacteria unclassified) in LPD and OTU0002 (*Spirochaetaceae*) in the SPD samples as taxonomic biomarkers associated with increasing *Yucca schidigera* ROI. OTU0004 sequence counts decreased, and OTU0005 sequence counts increased with ROI. BLASTn classified OTU0004 as *Methanobrevibacter millerae* strain ZA-10 with 99% sequence identity and OTU0005 as *Gilliamella intestini* strain LMG 28358 with 85% sequence identity, with the latter most likely to have been misclassified and thus representing a novel species. Previous studies have reported a related unclassified Gammaproteobacteria OTU with a close phylogenetic relationship to cultured strains in the succinate-producing family *Succinivibrionaceae* (2). Despite this, succinate was not detected in rumen samples in the current study. However, succinate is readily metabolized to propionate by rumen *Succiniclasticum* species (42), such as by OTUs 0001 and 0007 detected in the present study (File S2). This could provide a possible mechanism driving the increased propionate concentration associated with *Y. schidigera* extract ROI. Succinate and propionate production in the rumen utilize ruminal hydrogen (H_2_) that would otherwise be available for the reduction of CO_2_ to methane by the rumen archaea (43). This mechanism, and the relatively high proportion of *Succinivibrionaceae* species, has also been implicated in the low enteric methane emissions from Tammar wallabies (44). The combined effect of increased succinate and propionate, reduced H_2_ availability, and a decrease in the *Methanobrevibacter* biomarker (OTU0004) could indicate reduced methanogenesis as a result of supplementing *Yucca schidigera* extract in the present study. Reduced methane production with *Y. schidigera* extract supplementation has also been reported previously from *in vitro* studies (45) and in dairy cows, albeit at a high ROI (45 g/kg of substrate DM [46]). Rumen methane was not measured in the present study, but the Archaea:Bacteria ratio, which has been reported previously to be an effective proxy of methane production in beef cattle (g/kg DMI) (47), was not found to be affected.

Univariate analysis of normalized filtered sequence counts highlighted differences in the abundance of OTUs classified to *Fibrobacteraceae* and *Prevotellaceae* with *Y. schidigera* extract ROI, particularly at 15 g/day in both LPD and SPD samples. This effect on individual species reflected the broader result of the F:B ratio by ROI. Species of *Fibrobacteraceae,* such as *Fibrobacter succinogenes* S85 (e.g., OTU00023), are specialized fiber degraders in the rumen (48), whereas the Prevotellaceae, such as *Prevotella ruminicola* strain Bryant 23 (e.g., OTU0009), while not involved in fiber degradation, have roles in non-cellulose-carbohydrate and N metabolism (49, 50). The importance of rumen ammonia N for microbial growth has been mentioned previously; therefore, the potential of reduced rumen ammonia N with a higher ROI of *Y. schidigera* extract could come at the expense of the growth of some species of fibrolytic bacteria. Cultured *Prevotella* strains have also been characterized for their ability to ferment saponins (51), and species such as *Prevotella ruminicola* 23 have been reported to be more resilient to ammonia N reduction, with the ability to shift its transcription profile as a response to the availability of N (49).

### Performance and intake

The addition of *Yucca schidigera* to the TMR did not affect intake or milk yield at any of the ROI. There was, however, a linear increase in the proportion of milk fat concentration (g/kg). In previous studies, this effect was not detected (13), although the *Yucca schidigera* extract was supplemented at only 5 g/cow/day. Milk fat has been found to be positively correlated to the Firmicutes to Bacteroidetes ratio in the rumen microbiome (52). In the present study, the F:B was reduced at higher ROI (15 g/day), which was, in turn, associated with the higher milk fat concentration. The results for the F:B ratio were complicated by an interaction of ROI with the time of day relative to feed out. This, along with reduced ammonia concentration and the dependency of availability for microbial uptake on pH, could also influence the F:B ratio (53). Coincidentally, at 15 g/day ROI, liveweight and BCS were reduced linearly, although it is unclear if these factors are related.

### Conclusions

Adding *Yucca schidigera* extract as a dietary supplement to dairy cows altered rumen fermentation, the microbiome, and the performance of dairy cows. The reduction in pH variability could be of benefit in reducing subacute ruminal acidosis. Rumen ammonia N was also decreased with increasing inclusion rate, although the mechanism is not clear. The reduction in rumen ammonia could affect microbial protein synthesis in selected groups, possibly reducing fibrolytic species while benefiting more versatile species. Individual methanogenic Archaeal species may be reduced at higher rates of inclusion, although this was not reflected in the ratios of functional microbial groups involved in methanogenesis, and the effects on methane production are therefore unclear.

## Data Availability

Sequence data are available from the European Nucleotide Archive (www.ebi.ac.uk/ena) under project accession number PRJEB58908.

## SUPPLEMENTAL MATERIAL

Supplemental legends spectrum.00641-25-s0001.docx Legends for additional files and NMDS ROI supplementary files

Diet composition spectrum.00641-25-s0002.docx Diet composition and analyzed chemical composition of the basal diet.

NMDS ROI spectrum.00641-25-s0003.tif NMDS plot of microbiome beta diversity by *Y. schidigera* ROI.

Sequence analysis shell script spectrum.00641-25-s0004.txt Step by step processing of raw sequence data, including settings for quality control, clustering, and taxonomic classification.

OTU table spectrum.00641-25-s0005.xlsx OTU abundance, taxonomy, and representative sequences.

ASM does not own the copyrights to Supplemental Material that may be linked to, or accessed through, an article. The authors have granted ASM a non-exclusive, world-wide license to publish the Supplemental Material files. Please contact the corresponding author directly for reuse.
