# Supplementary material for: The rumen microbiota and metabolism of dairy cows are affected by the dietary rate of inclusion of Yucca schidigera extract
Source: Microbiol Spectr. 2025 Jun 12;13(8):e00641-25. doi: 10.1128/spectrum.00641-25 (PMC12323574; doi:10.1128/spectrum.00641-25)
Supplement: Supplemental legends — Legends for additional files and NMDS ROI supplementary files [file spectrum.00641-25-s0001.docx]

**Supplementary materials legends**

Additional File 1. Text file of sequence analysis shell script for parsing of raw sequence data with MOTHUR into a table of operational taxonomic units (OTUs) based on 97% sequence identity. Stages include sequence quality control settings, denoising, chimera removal alignment, clustering, sub sampling and taxonomic classification.

Additional File 2. Excel file showing operational taxonomic units for each sample, sub-sample normalised sequence abundance, representative sequence, taxonomic classification and sample metadata.

NMDS ROI. Non-metric Multi-dimensional Scaling (NMDS) plot of microbial communities from samples by rate of inclusion (ROI) *Yucca schidigera*. Stress = 0.17. PERMANOVA 10000 iterations: F-value: 1.25; R-squared: 0.043; P = 0.195.
