# Supplementary material for: The rumen microbiota and metabolism of dairy cows are affected by the dietary rate of inclusion of Yucca schidigera extract
Source: Microbiol Spectr. 2025 Jun 12;13(8):e00641-25. doi: 10.1128/spectrum.00641-25 (PMC12323574; doi:10.1128/spectrum.00641-25)
Supplement: Diet composition — and analyzed chemical composition of the basal diet. [file spectrum.00641-25-s0002.docx]

**Supplementary Table**. Diet composition and analysed chemical composition of the basal diet used to determine the effect of level of inclusion of *Yucca schidigera* extract on the performance, rumen metabolism, microbiome and N balance in dairy cows

| **Ingredient** | **kg/kg DM** |
| --- | --- |
| Grass silage | 0.223 |
| Maize silage | 0.360 |
| Barley | 0.103 |
| Faba Fibre^1^ | 0.051 |
| NovaPro^2^ | 0.097 |
| Rapeseed meal | 0.082 |
| Wheat distiller dark grains | 0.047 |
| Cane molasses | 0.009 |
| Urea (feed grade) | 0.004 |
| Megalac^3^ | 0.015 |
| Minerals/vitamins^4^ | 0.006 |
| Salt | 0.002 |
| **Total** | 1.000 |
|  |  |
|  |  |
| **Chemical analysis** |  |
| Dry matter, g/kg | 349 |
| Crude protein, g/kg DM | 175 |
| NDF, g/kg DM | 439 |
| Ash, g/kg DM | 73 |
|  |  |

^1^KW Alternative Feeds, Leeds, UK

^2^Trident Feeds, Peterborough, UK

^3^Volac, Royston, UK

^4^KW Alternative Feeds, Leeds, UK. Contained major minerals (g/kg): Ca, 220; P, 30, Mg, 80; trace minerals (mg/kg): Cu 1000; Zn, 3000, Mn 4000; Co, 80; I, 400; Se, 30; Vitamin A; 1,000,000 IU; Vitamin D3; 30,000 IU; All-rac alpha-tocopherol, 4000 IU; Vitamin B_12_, 2500µg; Biotin, 135 mg.
