# Supplementary figures and images for: The rumen microbiota and metabolism of dairy cows are affected by the dietary rate of inclusion of Yucca schidigera extract
Source: Microbiol Spectr. 2025 Jun 12;13(8):e00641-25. doi: 10.1128/spectrum.00641-25 (PMC12323574; doi:10.1128/spectrum.00641-25)

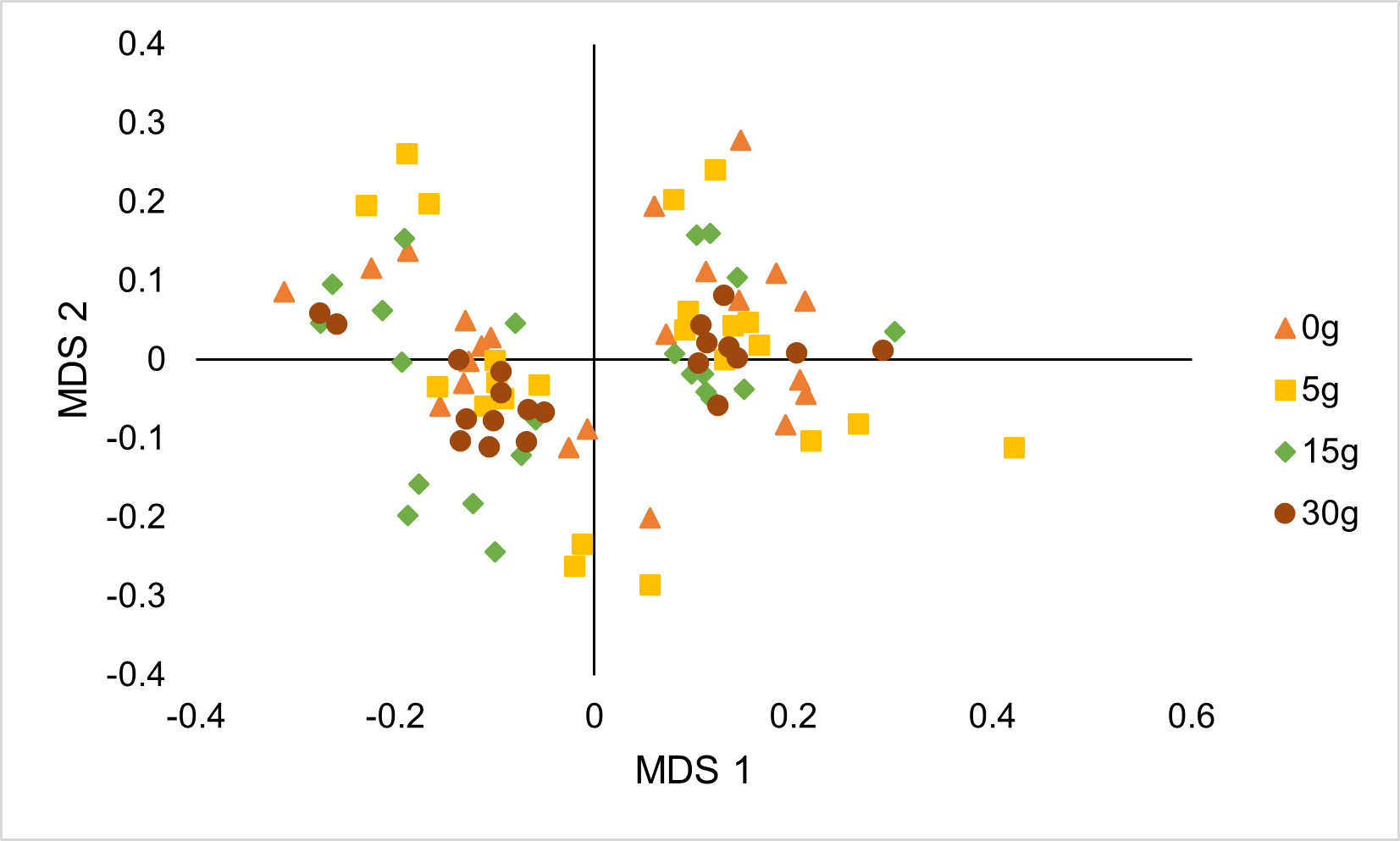

Supplement: NMDS ROI — NMDS plot of microbiome beta diversity by Y. schidigera ROI. [file spectrum.00641-25-s0003.tif]
